# Analysis of Insecticide Resistance-Related Genes of the Carmine Spider Mite *Tetranychus cinnabarinus* Based on a *De Novo* Assembled Transcriptome

**DOI:** 10.1371/journal.pone.0094779

**Published:** 2014-05-15

**Authors:** Zhifeng Xu, Wenyi Zhu, Yanchao Liu, Xing Liu, Qiushuang Chen, Miao Peng, Xiangzun Wang, Guangmao Shen, Lin He

**Affiliations:** Key Laboratory of Entomology and Pest Control Engineering of Chongqing, College of Plant Protection, Southwest University, Chongqing, China; Instituto de Biotecnología, Universidad Nacional Autónoma de México, Mexico

## Abstract

The carmine spider mite (CSM), *Tetranychus cinnabarinus*, is an important pest mite in agriculture, because it can develop insecticide resistance easily. To gain valuable gene information and molecular basis for the future insecticide resistance study of CSM, the first transcriptome analysis of CSM was conducted. A total of 45,016 contigs and 25,519 unigenes were generated from the *de novo* transcriptome assembly, and 15,167 unigenes were annotated via BLAST querying against current databases, including nr, SwissProt, the Clusters of Orthologous Groups (COGs), Kyoto Encyclopedia of Genes and Genomes (KEGG) and Gene Ontology (GO). Aligning the transcript to *Tetranychus urticae* genome, the 19255 (75.45%) of the transcripts had significant (e-value <10^−5^) matches to *T. urticae* DNA genome, 19111 sequences matched to *T. urticae* proteome with an average protein length coverage of 42.55%. Core Eukaryotic Genes Mapping Approach (CEGMA) analysis identified 435 core eukaryotic genes (CEGs) in the CSM dataset corresponding to 95% coverage. Ten gene categories that relate to insecticide resistance in arthropod were generated from CSM transcriptome, including 53 P450-, 22 GSTs-, 23 CarEs-, 1 AChE-, 7 GluCls-, 9 nAChRs-, 8 GABA receptor-, 1 sodium channel-, 6 ATPase- and 12 Cyt b genes. We developed significant molecular resources for *T. cinnabarinus* putatively involved in insecticide resistance. The transcriptome assembly analysis will significantly facilitate our study on the mechanism of adapting environmental stress (including insecticide) in CSM at the molecular level, and will be very important for developing new control strategies against this pest mite.

## Introduction

Carmine spider mite (CSM), *Tetranychus cinnabarinus*, also known as cotton red spider, belongs to Class Arachnida, Subclass Acari, Order True Acarina, Family Tetranychidae [1], [2]. It is one of the most damaging pest mites in agriculture and forestry. The CSM mainly distributes in warm regions of the world and utilizes stylet to suck plant sap, causing mechanical damage to the host tissue [3], [4]. Serious infestation of CSM might cause leaves to dry off loss water or even die, causing severe economic losses. It parasitize more than one hundred plant species, such as cotton, various vegetables, melons, beans, roses, jujube, Chinese herbal medicine and many other economic crops and ornamental plants, leading to significantly reduced quality and yield [2], [5].

CSM and two-spotted spider mite (TSM), *Tetranychus urticae* Koch, are both widely distributed polyphagous pest mites. Both of them are polymorphic in morphology, and are very similar in external morphologies. In different hosts and different regions, these two species present obvious similarities in external morphology. Therefore, many researchers considered them as two forms (red and green) of a single species (*Tetranychus urticae*) [6]–[12]. In 1956, Boudreaux [13] first separated CSM from TSM as an independent species based on experimental results of breeding and morphological characteristics. In 1990, Kuang [14] further confirmed that CSM and TSM were two entirely different species with complete reproductive isolation by performing a comprehensive comparative study of the two species, focusing on the aspects of hybridization, changes in body color, body size, external morphological features, ultrastructure, physiology and biochemistry, and ecology. So far, many taxonomists still questioning the two species just were red and green forms of *T. urticae* [15]–[21]. Therefore, the published genomic information of TSM [22] could not be fully utilized when investigating CSM.

Currently, the control and prevention of CSM mainly depends on spraying chemical insecticides and acaricides. However, due to its characteristics such as small individual size, strong reproductivity, short developmental duration, high inbreeding rate, frequent opportunities of receiving insecticide, strong adaptability and high mutation rate, this species of mite can easily develop insecticide resistance [1], [23]. Insecticide resistance is a micro-evolutionary phenomenon, and the enhanced resistant capability selected by insecticides is hereditary. At the molecular level, there are two mechanisms underlying the insecticide resistance in arthropods, namely enhanced insecticide metabolism and reduced sensitivity of targets to insecticides [24]. However, the lack of genetic information of CSM limits our ability to understand the mechanisms of insecticide resistance development, preventing us from developing effective resistance management strategies.

The three main targets for commonly used insecticides are ligand-gated ion channels, voltage-gated ion channels and acetylcholinesterase [25]. Currently, the most frequently studied ligand-gated ion channels include the nicotinic acetylcholine receptor (nAChR), gamma-aminobutyric acid (GABA) receptor and glutamate-gated chloride channels (GluCls). The most frequently studied voltage-gated ion channels is sodium channels. In addition, cytochrome b (Cyt b) is a new target, which act as an alternative target of acaricide, bifennazate [26], [27]. Metabolic resistance is generally associated with enzymes encoded by multiple gene families including cytochrome P450, carboxylesterases (CarEs) and glutathione S-transferase (GSTs).

Prior to this study, the NCBI database only contains 122 nucleotide sequences and 97 amino acids sequences of CSM. These genetic data are not enough to elucidate the mechanisms of insecticide resistance development and gene regulation of CSM from the molecular level. Investigating the gene sequences by traditional biological methods is often time-consuming and inefficient. However, the emergence of high-throughput sequencing technology provides researchers with a fast and low-cost way to obtain genetic data.

Transcriptome is the complete repertoire of mRNAs transcribed by a living cell, i.e. the sum of genetic information transcribed from the genomic DNA. Investigation of transcriptome is an important approach to study phenotypes and functions of cells. In order to obtain the information of transcribed genes of CSM, especially the genes that involved in the development of insecticide resistance, we employed the high-throughput sequencing platform-Illumina HiSeq™ 2000 to complete the whole transcriptome sequencing of the CSM. Based on the transcriptome analysis, several categories of genes that might be involved in metabolic and target resistance were analyzed.

## Results and Discussion

### *De novo* Transcriptome Assembly of CSM

**Figure 1:**
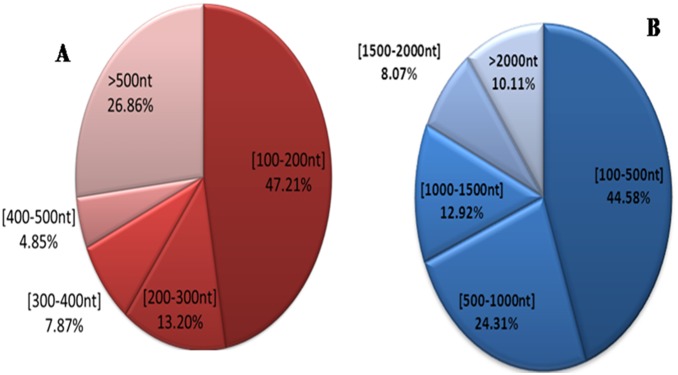
A, Length distribution charts of contigs. B, Length distribution charts of unigenes.

The CSM transcriptomes were generated from four life stages (egg, larva, nymph and adult) of CSM via the Illumina sequencing. We then constructed a mass gene database that contains a total of 4,687,231,140 nucleotides (nt) and 54,350,230 reads, each of which was approximately 90 base pair (NCBI: SRA052165). After eliminating low quality reads from the raw reads, there were 52,080,346 clean reads remained, which accounted for 95.82% of the raw reads. All clean reads were assembled, compared and ligated using the short reads assembly software Trinity, so as to get the contigs and unigenes from the CSM transcriptome. These reads were assembled into 25,519 unigenes, which had been submitted to BioProject with accession number PRJNA210716 in the NCBI-database and the basic statistics for the transcriptome dataset were shown in Table 1. The 47.21% of contigs ranged from 100 to 200 nt and 26.86% contigs were more than 500 nt in length (Figure 1-A). The 44.58% of unigenes ranged from 100 to 200 nt, 24.31% ranged from 500 to 1000 nt and 31.10% were more than 1000 nt in length (Figure 1-B).

**Table 1:** Statistics of assembled sequences.

Raw data		
Number of reads	54,350,230	
**Clean data**		
Number of reads	52,080,346	
Total Length(nt)	4,687,231,140	
Q20 percentage	98.70%	
N percentage	0.00%	
GC percentage	40.20%	
**After assembly**		
	**Contigs**	**Unigenes**
Number	45,016	25,519
Total Length(nt)	22,683,370	23,907,206
Mean length(nt)	504	937
N50	1127	1485
Total Consensus Sequences	-	25,519
Distinct Clusters	-	237
Distinct Singletons	-	25,282

### Sequencing Accuracy of the CSM Transcriptome

Ten complete mRNA sequences of CSM were chosen randomly from the NCBI nucleotide database to evaluate the sequence accuracy of transcriptome assembly. Sequence alignment was performed for 10 chosen full-length mRNA sequences and 23 corresponding annotated unigenes from the transcriptome assembly, in which 95% identity and 80% alignment length were set as thresholds. The average identity between the 10 previously identified CSM cDNA sequences in the NCBI nucleotide database and the 23 assembled sequences identified in the CSM transcriptome was 99.21% (Table 2), which is similar to the level of sequencing accuracy reported by other studies for Illumina technology [28], [29]. Sequencing error or nucleotide polymorphisms may be responsible for the nucleotide diversity between assembled sequences and the submitted sequences in the NCBI nucleotide database.

**Table 2:** The information of the alignment between the unigenes from transcriptome and genes from NCBI.

Gene ID or gene name	Start	End	Identity (%)	Alignment length (bp)	Length (bp)
(GU196305) voltage-gatedsodium channel protein	-	-	-	-	5246
Unigene1006_A	128	403	100	274	276
Unigene2178_A	1	370	100	370	370
Unigene5257_A	1	230	96	230	230
Unigene5615_A	1	428	99	428	428
Unigene5926_A	1	218	100	218	218
Unigene23995_A	1	483	100	483	483
Unigene24821_A	1	321	100	321	321
**(**EU130462**)**esterase TCE2	-	-	-	-	2015
Unigene9913_A	1	1822	100	1822	1822
Unigene9914_A	90	1772	98	1659	1682
**(**EU130461**)**esterase TCE1	-	-	-	-	1786
Unigene10628_A	115	1815	100	1700	1700
**(**HM753535**)**mitochondrion,complete genome	-	-	-	-	13092
Unigene3089_A	2	578	100	577	577
Unigene9856_A	16	1396	99	1381	1381
Unigene21798_A	1	459	99	459	459
Unigene9944_A	1	830	99	830	830
Unigene8511_A	1	698	99	698	702
Unigene11811_A	1	426	99	426	426
Unigene15356_A	1	305	99	305	305
**(**DQ310791**)** heat shockcognate 70	-	-	-	-	2275
Unigene4399_A	1	232	98	232	232
**(**EU679413**)** heat shockprotein 70–2	-	-	-	-	2305
Unigene9282_A	1	2259	99	2259	2259
**(**EU851046**)** heat shockprotein 90	-	-	-	-	2591
Unigene9300_A	1	2584	99	2584	2584
**(**EU679412**)** heat shockprotein 70–1	-	-	-	-	2446
Unigene9813_A	1	2405	99	2405	2405
**(**EU679414**)** heat shockprotein 70–3	-	-	-	-	2284
CL75.Contig1_A	1	2239	99	2239	2239
**(**EU362111**)** GABAreceptor alpha subunit	-	-	-	-	1730
Unigene12658_A	1	769	100	769	772
Unigene15264_A	283	1154	100	872	1154

**Note:** Start and End indicated the starting and ending position of the alignment between unigenes from transcriptome and the genes from NCBI nucleotide database, respectively.

### Genome Mapping Results to *T. Urticae*

Since there is no genome sequence available for CSM, unambiguous sequence alignment of the transcripts to a reference Tetranychidae genome could provide additional measures of the transcriptome assembly accuracy and completeness. The assembled sequences were mapped to the *T. urticae* genomes. Aligning the transcript to *T. urticae* genome, the 19255 (75.45%) transcripts had significant (e-value <10^−5^) matches to *T. urticae* genome database (Table S1). Transcripts that did not map to the *T. urticae* genome (24.55%), as well as partial alignments may represent mis-assemblies in the transcriptome or the genome, rapidly evolving genes or the rapid evolution of untranslated regions (UTRs) [30].

**Figure 2:**
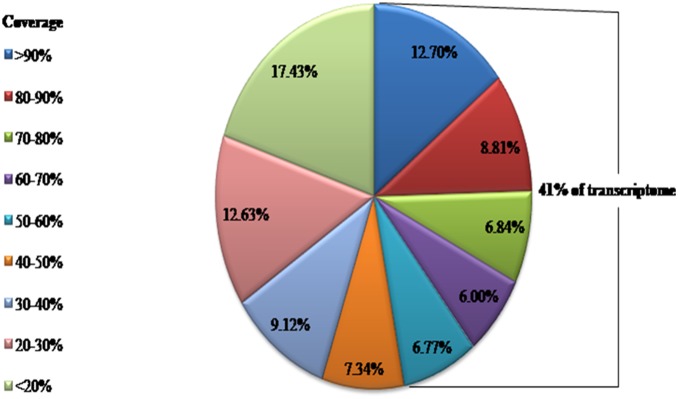
The distribution of the CSM transcripts in different coverage that aligned to *T. utricae* genome.

As a starting point for transcript analysis, the proportion of the CSM transcriptome that was homologous to a predicted protein sequence in *T. urticae* genomes was analyzed. Protein similarity to *T. urticae* proteomes was assessed using BLASTX (e-value threshold of 1.0E-5). A total of 19,111 sequences (74.89%) in our dataset had a significant match with *T. urticae* (Table S2). To further validate the accuracy of our ortholog assignment, an analysis of the protein length coverage for BLASTX alignments showed a considerable proportion of transcriptome with mostly coverage (average protein length coverage was 42.55%) of their corresponding *T. urticae* match, with 41% of the orthologs covering more than half of their corresponding *T. urticae* matches (Figure 2).

In summary, transcript mapping to reference genomes revealed a degree of incompleteness when using *T. urticae* as our reference. Incomplete CSM transcriptome could underestimate the diversity of protein configurations and thus, may limit protein identification by proteomic approaches in the absence of the genome. Despite the limitations in our dataset, the transcriptome genes information will be useful for experimentalists when designing primers and probes for one gene-targeted analysis, especially when combined use with the *T. urticae* genome, the researchers will be very convenient and fast obtaining a large number of *T. cinnabarinus* target genes with partial or full sequences.

### CEGMA Analysis

To assess the completeness of the transcriptome assembly, the CEGMA (Core Eukaryotic Genes Mapping Approach) software was applied to identify the presence of a core protein set consisting of 458 highly conserved proteins that are found in a wide range of eukaryotes [31]. This software is usually used to assess the completeness of a genome assembly, but should also enable the assessment of a transcriptome under different interpretations [32]. We identified 435 core eukaryotic genes (CEGs) in the CSM dataset corresponding to 95% coverage (Table S3), which is slightly lower than *T. urticae* genome (448 of 458 CEGs, 98%). Considering the transcriptome for CSM showed a higher coverage than that for *Anopheles albimanus* (showed 90% coverage) and the coverage ranged from 95–98% in the other genome sequenced species [30], [32], we could say that the quality of transcriptome assembly for CSM is considerably good.

### Unigene Functional Annotation by Nr, GO, COG, and KEGG

**Figure 3:**
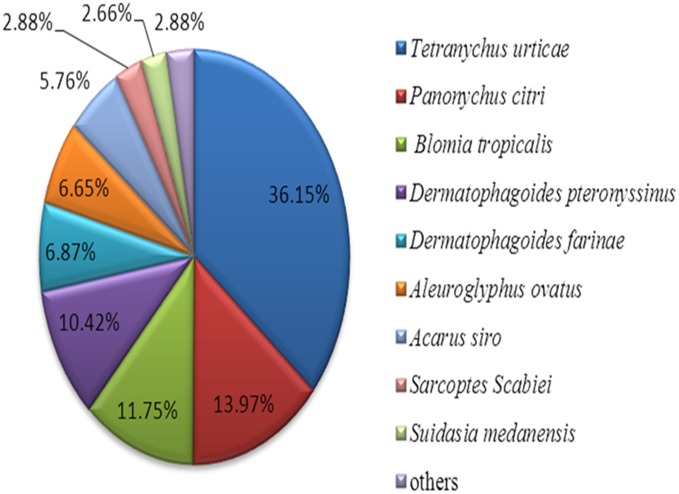
Species distribution of homology search of unigenes against the Nr database. The species distribution is shown as a percentage of the total homologous sequences in the NCBI Nr protein database with an E-value <10^−5^.

A total of 14,589 unigenes (57.17%) from the CSM transcriptome returned an above cut-off blast hit to the NCBI non-redundant protein database. The species distribution of the top blastx hits for each unique sequence was shown in Figure 3. The unambiguous assembled sequences revealed that the greatest number of matches (36.15%) was from *T. urticae*, followed by *Panonychus citri* (13.97%), *Blomia tropicalis* (11.75%), and *Dermatophagoides pteronyssinus* (10.42%).

**Figure 4:**
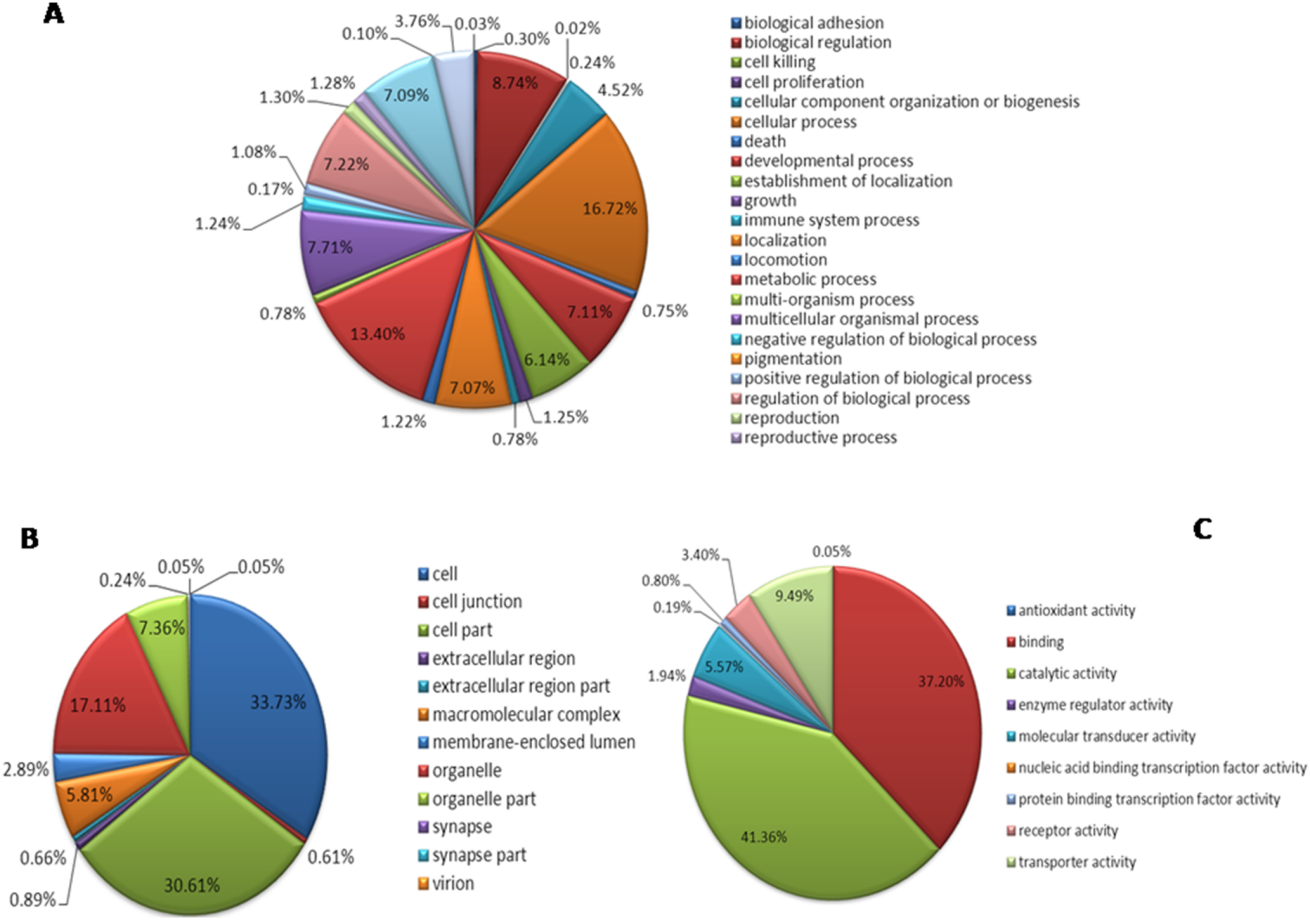
Gene Ontology annotation and classification of the CSM transcriptome. A: Biological process B: Cellular component C: Molecular function.

Based on the CSM transcriptome assembly, 2,447 (16.13%) sequences were annotated in the GO database, which were divided into a total of 47 groups in three ontology categories (molecular function, cellular component, biological process). The “molecular function” ontology category contains 26 groups. The largest group is “cellular process” with 1,054 unigenes, and the smallest group is “cell killing” with only one unigene (Figure 4-A). The “cellular component” ontology category contains 12 groups. The largest group is “cell” with 1,435 unigenes, and the smallest groups are “virion” and “synapse part”, with only two unigenes in each group (Figure 4-B). The “biological process” ontology category contains 9 groups. The largest group is “catalytic activity” with 876 unigenes and the smallest group is “antioxidant activity” with only one unigene (Figure 4-C).

**Figure 5:**
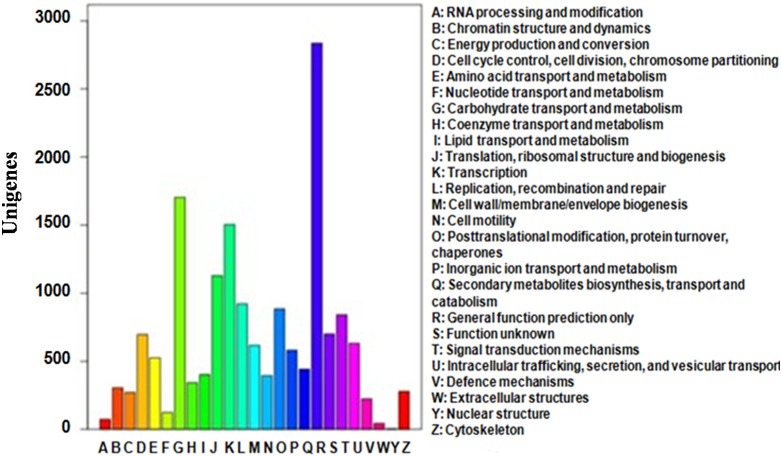
Distribution of COG functional annotation of the CSM transcriptome.

In order to annotate the detail function of genes, COG database was used. In total, 6,558 unigenes (43.24%) were annotated and these genes were divided into 25 categories. A total of 2,834 unigenes, which was approximately half of the 6,558 unigenes, were placed into the “General function prediction only” category. Followed by “Carbohydrate transport and metabolism” (1,703, 25.97%), “Transcription” (1,504, 22.93%), and “Translation, ribosomal structure and biogenesis” (1,129, 17.22%). The smallest one is “Nuclear structure” with only four unigenes, accounting for only 0.06% of the functionally annotated unigenes (Figure 5).

To identify the genes that involved in metabolic pathways, a total of 11,545 unigenes (45.24%) were mapped to the KEGG pathway database [33]. These unigenes were divided into 241 pathways. The largest pathway is “Metabolic pathways” with 1,529 unigenes, accounting for 13.24% of the unigenes with functional annotation, followed by “Pathways in cancer” (439, 3.80%) and “Lysosome” (400, 3.46%) (Table S4). The smallest pathways are “Allograft rejection”, “D-Arginine and D-ornithine metabolism” and “Graft-versus-host disease” with only one unigene in each group, only accounting for 0.01% of the unigenes with functional annotation.

### Identification of Genes Related to Insecticide Resistance

Based on the results of nr blastx, the unigenes that possibly involved in insecticide resistance development in the CSM transcriptome assembly were selected manually. After eliminating redundant and shorter sequences, we identified 3 categories of genes that associated with metabolic resistance (Cytochrome P450, CarEs and GSTs) and 7 categories of genes that associated with target resistance (GABA receptor, AChE, sodium channel, GluCls, ATPase, nAChRs and Cytb) (Table 3). The genes in these categories have been proved involved in insecticide resistance development in arthropod.

**Table 3:** The statistic information for special unigenes associated with insecticide resistance.

Unigene Category	Transcripts Number	Unigene Number	Maximum unigene length	Minimum unigene length	Average length
P450	81	53	1542	213	803
GST	27	22	732	201	532
CarE	29	23	5850	630	3105
IGluRs	17	7	855	234	454
nAChRs	18	9	1431	213	470
GABA receptor	12	8	1152	207	613
sodium channel	7	1	1095	216	435
AChE	1	1	6183	6183	6183
ATP synthase	14	6	3747	282	2127
Cyt b	12	12	996	288	604

### Three Categories of Genes Mediating Metabolic Resistance

**Figure 6:**
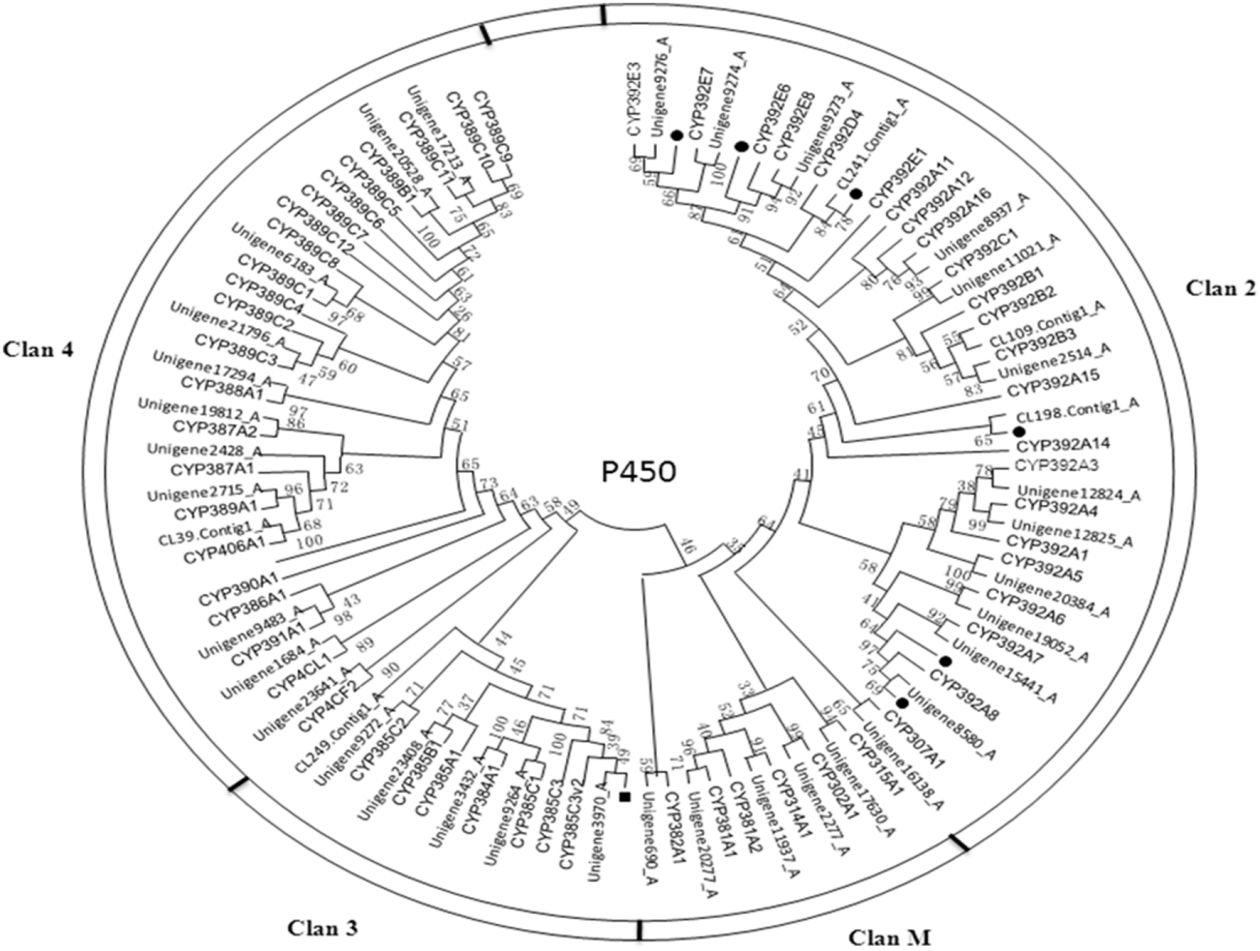
Phylogenetic tree of cytochrome P450 from *T. cinnabarinus* and *T. urticae* (Tu). The tree was constructed from the multiple alignments using PhyML3.1 software and generated with 500 bootstrap trials using maximum likelihood approach method.

The cytochrome P450 family is a major family of enzymes functioning in detoxification and metabolism [25]. Because of the genetic diversity, broad substrate specificity, and catalytic versatility, P450s can mediate resistance to all classes of insecticides [34]. In present study, a total of 81 CSM P450 transcripts were identified in the dataset with an average length of 803 bp, and 53 unigenes were found from the 81 transcripts, which were further examined to confirm that each was respectively aligned to a certain *T. urticae* P450 protein sequence (Table S5). The reasons why the numbers of P450 genes are approximations in so far genome-sequenced species were provided by Feyereisen with details [35]. The approximate numbers for CSM P450 genes might be 80 between 90 with estimation, according to that in its sibling species *T. urticae* whose genome were sequenced is 86 (Table 4), and from this sense the probability that the present 81 transcripts obtained from CSM transcriptome represented 81 P450 genes could not be excluded. The number of P450 genes in arthropod varies widely (e.g., 36 in the human body louse *Pediculus humanus* to 160 in the Egyptian mosquito *Aedes aegypti*, Table 4), but so far all the CYP genes can be assigned to one of four clans: CYP2, CYP3, CYP4 and the mitochondrial CYP clan (CYP M) [36]. The mitochondrial clan in vertebrates is involved in essential physiological functions, such as metabolize sterols, steroids or secosteroids (vitamin D), but that in insect is involved in xenobiotic metabolism [37]. CYP2 clade in insect includes P450s with essential physiological functions, e.g. ecdysone metabolism and juvenile hormone biosynthesis [38]. Considerable evidence links members of CYP3 clade in insect to xenobiotic metabolism and also insecticide resistance, whereas some CYP4s appear to be inducible metabolizers of xenobiotics and others have been linked to odorant or pheromone metabolism [37]. Phylogenetic analysis showed that the majority of CSM P450s belongs to clan 2 (22) and clan 4 (17) compared to clan 3 (8) and clan M (6) (Figure 6). Interestingly, we found a “bloom” or family expansion in clade 2 and a contraction in clade 3 in the *Tetranychus* lineage compared to Insecta (Table 4). Considering members of the CYP3 and CYP4 clades in most insect species are commonly involved in environmental response/detoxifying functions against xenobiotics and phytotoxins [39], so the CYP2 and CYP4 clades exert these functions in *Tetranychus* mites quite possibly.

**Table 4:** Difference in the number of genes in different P450 families between the CSM transcriptome and genomes of other species.

Species	CYP2 clade	CYP3 clade	CYP4 clade	Mitochondrial CYPs	Total
**Insecta**					
*Trialeurodes vaporariorum*	3	34	13	7	57
*Acyrthosiphon pisum*	10	33	32	8	83
*Myzus persicae*	3	63	48	1	115
*Drosophila melanogaster*	6	36	32	11	85
*Anopheles gambiae*	10	42	45	9	106
*Aedes aegypti*	12	82	57	9	160
*Bombyx mori*	7	30	36	12	85
*Apis mellifera*	8	28	4	6	46
*Nasonia vitripennis*	7	48	30	7	92
*Tribolium castaneum*	8	72	45	9	134
*Pediculus humanus*	8	11	9	8	36
*Daphnia pulex*	20	12	37	6	75
**Acarina**					
*Tetranychus cinnabarinus*	22	8	17	6	53
*Tetranychus urticae*	48	10	23	5	86
*Ixodes scapularis*	39	120	46	0	205

**Figure 7:**
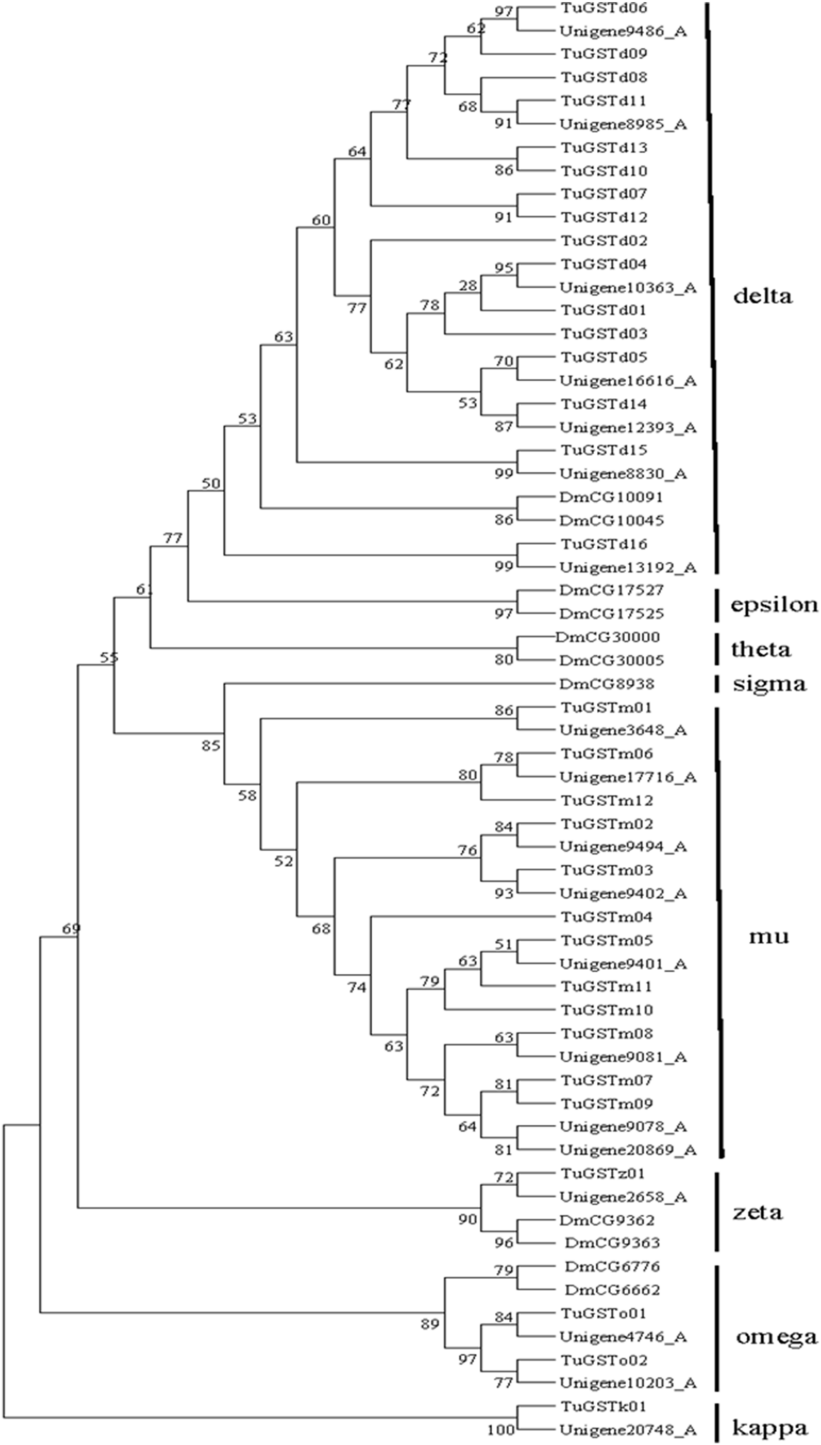
Phylogenetic tree of GSTs identified from *T. cinnabarinus*, *T. urticae* (Tu) and *D.melanogaster* (Dm). The tree was constructed from the multiple alignments using PhyML3.1 software and generated with 500 bootstrap trials using maximum likelihood approach method.

Glutathione S-transferases (GSTs) are a class of multifunctional detoxification enzymes and play an important role in the metabolism of a variety of insecticides, especially organophosphorus insecticides [40]. The increased expression and activity of GSTs has been documented as a mechanism of insect resistance [41], [42]. In our study, a total of 27 putative GSTs transcripts were identified in the CSM transcriptome (Table S6). Based on the results of the closest BLAST hits against the NCBI nr database, *T. urticae* genome database and phylogenetic analysis, 22 GSTs genes were remained and belong to five classes, mu (8), delta (7), omega (2), zeta (1) and kappa (1), unknown (3), respectively (Figure 7). The GSTs family and number of GSTs genes between Subclass Acari such as CSM and Insecta are different (Table 5). For example, the delta and epsilon GST classes in Insecta seem to be implicated in xenobiotic detoxification [38], but no epsilon GST class gene was found in the Acari (except only one was found in *Ixodes scapulari*) replaced by mu GST class which also was responsible for insecticides resistance [43]. The sigma GST class is widespread in Insecta but not identified in Acari, which was further evidenced playing roles in the flight muscle operating under oxidative stress [44]. Finally, 1 gene of the kappa class was found in 3 species of Acari but not in Insecta, which had high catalytic activity for aryl halides, such as CDNB, and can reduce CuOOH and (S)-15-hydroperoxy-5, 8, 11, 13-eicosatetraenoic acid [45], [46].

**Table 5:** Difference in the number of genes in different GSTs families between the CSM transcriptome and genomes of other species.

Species	alpha	delta	epsilon	mu	omega	pi	sigma	theta	zeta	unknown	kappa	total
**Insecta**												
*Trialeurodes vaporariorum*	-	9	1	-	-	-	5	-	1	-	-	16
*Acyrthosiphon pisum*	-	10	-	-	-	-	6	2	0	-	-	18
*Myzus persicae*	-	8	-	-	-	-	8	2	-	-	-	18
*Drosophila melanogaster*	-	11	14	-	5	-	1	4	2	-	-	37
*Anopheles gambiae*	-	12	8	-	1	-	1	2	1	3	-	28
*Apis mellifera*	-	1	-	-	1	-	4	1	1	-	-	8
*Nasonia vitripennis*	-	5	-	-	2	-	8	3	1	-	-	19
*Tribolium castaneum*	-	3	19	-	4	-	7	1	1	-	-	35
*Bombyx mori*	-	4	8	-	4	-	2	1	2	2	-	23
**Acarina**												
*Tetranychus cinnabarinus*	-	7	-	8	2	-	-	-	1	3	1	22
*Tetranychus urticae*	-	16	-	12	2	-	-	1	-	-	1	32
*Ixodes scapularis*	-	2	1	29	4	-	-	6	12	24	1	79

**Figure 8:**
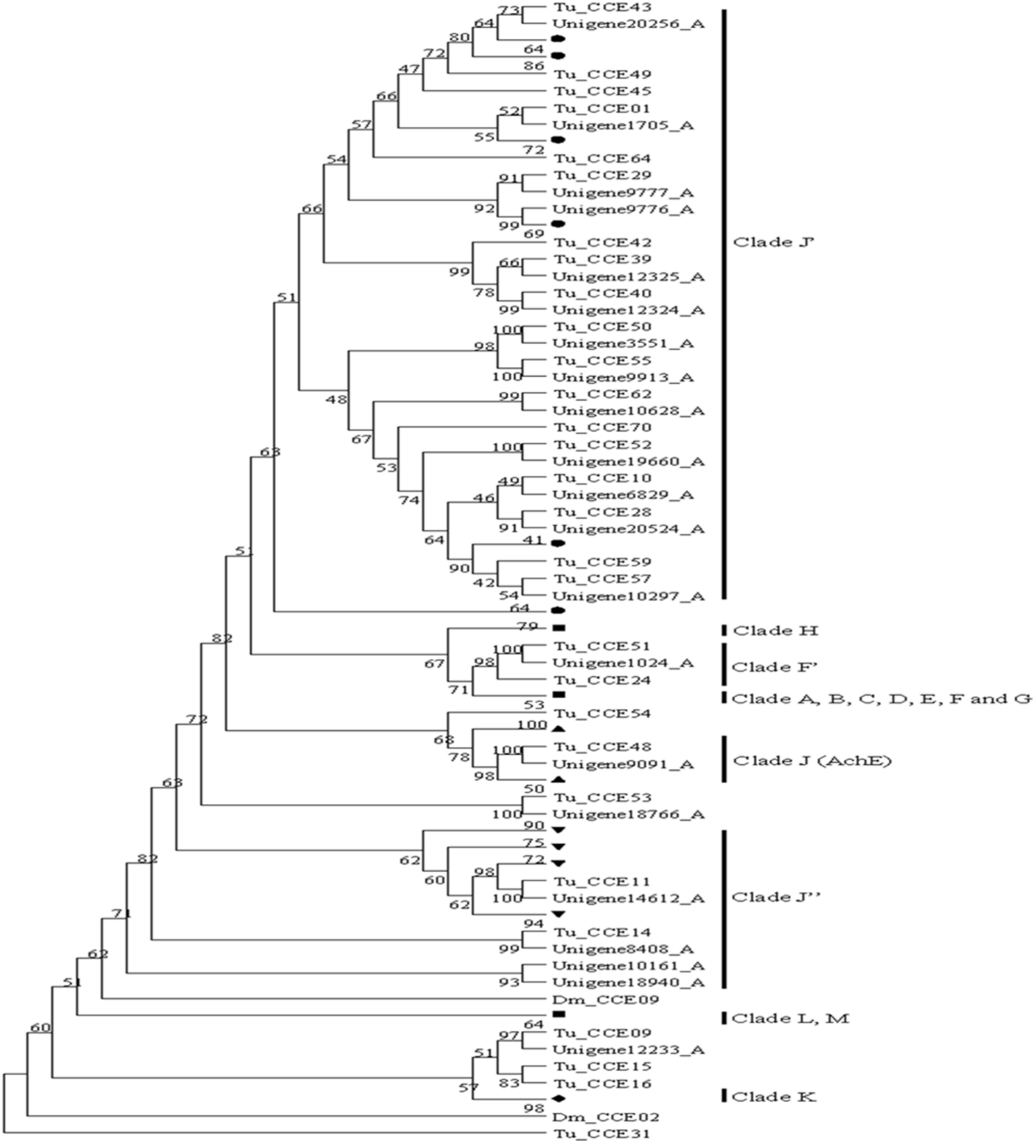
Phylogenetic tree of CCE identified from *T. cinnabarinus*, *T. urticae* (Tu), *A. gambiae* (Ag), *A. mellifera* (Am) and *D.melanogaster* (Dm). The tree was constructed from the multiple alignments using PhyML3.1 software and generated with 500 bootstrap trials using maximum likelihood approach method.

The main physiological functions of carboxylesterases (CarEs) include the degradation of the neurotransmitters, metabolism of specific hormones and pheromones, detoxification, defense and behavior. It is a hydrolase and can hydrolyze carboxyl ester bond and phosphodiester bond, thus metabolizing various ester bond-containing insecticides. Studies have shown that the amplification of CarEs genes is one of the important mechanisms that are involved in insecticide resistance [47]–[49]. Our study showed that 29 CarEs transcripts have been identified in the CSM transcriptome. After mapped these transcripts to the genome of *T. urticae*, a total of 23 CarEs sequences were confirmed to be unique genes (Table S7). Based on phylogenetic analysis with other known insects CCEs or the identification of closest blastn hits in the *T. urticae* genome database, CSM CarEs were divided into three clades, with 13 unique transcripts in Clade J', 4 in Clade J'', 1 in Clade F' and 5 in undetermined (1 in Clade J was AChE) (Figure 8). Comparative analysis of CCEs showed that the number of CCEs in Acari and Insecta is about the same, with the exception that *T. urticae* had a significant expansion, however, the majority of CCEs are assigned to the Neuro/developmental class in Acari. It is noteworthy that the dietary class of CCEs is widespread in insect but not found in the Acari (Table 6).

**Table 6:** Difference in the number of genes in different CCE families between the CSM transcriptome and genomes of other species.

	Dietary	Hormone/semiochemical	Neuro/developmental	Undetermined	Total
**Insecta**					
*Trialeurodes vaporariorum*	12	6	9	-	27
*Acyrthosiphon pisum*	5	18	7	-	30
*Myzus persicae*	5	12	5	-	22
*Apis mellifera*	8	5	11	-	24
*Drosophila melanogaster*	13	8	14	-	35
*Anopheles gambiae*	16	14	21	-	51
**Acarina**					
*Tetranychus cinnabarinus*	-	1	19	4	24
*Tetranychus urticae*	-	2	66	3	71
*Ixodes scapularis*	-	8	30	-	38

### Positive Selection Analysis of Genes Encoding Metabolic Enzyme

To identify metabolic enzyme encoding genes of *T. cinnabarinus* undergoing positive selection, a ω  =  dN/dS analysis in *T. cinnabarinus*/*T. urticae* ortholog pairs was performed. Generally speaking, synonymous and nonsynonymous substitution rates are defined under the comparison of two DNA sequences, namely dS and dN represent the numbers of synonymous and nonsynonymous substitutions per site, respectively. Thus, the ratio ω measures the difference between the two rates and is most easily understood from a mathematical description of a codon substitution model. In other words, an amino acid in neutral change will be fixed at the same rate as a synonymous mutation with ω = 1, in deleterious change will reduce its fixation rate, thus ω<1, in selective advantage change with ω>1. Therefore, significant advantage change offers convincing evidence for diversifying selection [50]. Among the 34 pairs of *T. cinnabarinus*/*T. urticae* orthologs ω values ranged from 0.910 to 2.595, with an average of 1.513, in which 32 pairs had a ω value greater than 1 (Table 7), suggesting these 32 unigenes were under positive selection.

**Table 7:** Summary of dN/dS analysis.

UnigeneID	Tu ID ortholog	ω dN/dS	Length of alignmentsequence (nt)
**P450s**			
Unigene20528_A	tetur25g02060	1.124	1539
Unigene17294_A	tetur03g05190	2.357	1516
Unigene2715_A	tetur25g02050	1.499	1515
Unigene9483_A	tetur36g00920	1.307	1671
Unigene23641_A	tetur09g03800	1.205	576
Unigene3432_A	tetur38g00650	1.664	1491
Unigene9264_A	tetur26g01470	1.326	1491
Unigene11937_A	tetur03g03020	1.729	879
Unigene2277_A	tetur05g02550	1.300	1539
Unigene17630_A	tetur06g05620	1.300	1956
Unigene15441_A	tetur16g03500	2.526	540
Unigene19052_A	tetur11g00530	1.604	1518
Unigene20384_A	tetur11g04390	1.383	1275
Unigene12825_A	tetur07g06480	1.383	1023
Unigene11021_A	tetur03g03950	1.770	1227
Unigene8937_A	tetur06g04520	1.151	1476
Unigene9273_A	tetur27g00350	1.140	1509
Unigene9274_A	tetur27g00340	1.140	1044
**CCEs**			
Unigene9777_A	tetur11g01500	1.607	939
Unigene12324_A	tetur16g02390	1.668	1016
Unigene3551_A	tetur20g03250	2.455	1133
Unigene9913_A	tetur26g0113	1.124	1820
Unigene10628_A	tetur30g01290	1.539	1852
Unigene19660_A	tetur24g01310	2.595	2070
Unigene1024_A	tetur23g00910	1.335	1420
Unigene9091_A	tetur19g00850	1.292	2775
Unigene14612_A	tetur01g14180	1.496	1899
Unigene8408_A	tetur02g04030	1.040	1987
Unigene12233_A	tetur01g14090	0.910	1974
**GSTs**			
Unigene8985_A	tetur26g01490	1.414	657
Unigene10363_A	tetur01g02510	1.515	648
Unigene13192_A	tetur31g01390	1.597	642
Unigene3648_A	tetur03g09230	1.974	672
Unigene20748_A	tetur22g02300	0.963	687

### Seven Categories of Genes Mediating Target Resistance

Glutamate-gated chloride channels (GluCls), also known as inhibitory glutamate receptors (IGluRs), belong to the superfamily of cysteine loop ligand-gated ion channel, and the function of Glucls is mainly in mediating inhibitory neurotransmission in nerve and muscle cells [51]. Because of GluCls are found only in invertebrates and have not been found in vertebrates, it is the ideal target for highly selective insecticides. Based on electrophysiological and pharmacological studies, glutamate receptors are divided into two categories termed ionotropic and metabotropic receptors. Insecticides that act on GluCls include abamectin, ivermectin, fipronil and the indole diterpenoid compound nodulisporic acid. A particular mutation in the α-subunit of GluCls causes the substitution of one amino acid, resulting in reduced sensitivity of the mutant channel to insecticide and thereby causing insecticide resistance [52]. A total of 7 GluCls sequences were identified from the CSM transcriptome (Table S8), but most of insects, such as *D. melanogaster*, *T. castaneum* and *A. mellifera*, only have one glutamate-gated chloride channel subunit.

The nAChRs represent a diverse family of cys-loop ligand-gated ion channels. It plays an important role in the transmission of nerve signals at the postsynaptic membrane in both vertebrates and invertebrates [53]. The current insecticides that are acting on insect nAChR mainly include nereistoxin, neonicotinoid and the biological insecticide, spinosad. These insecticides specifically bind to the insect nAChR and block the normal neural function of the receptors, thus leading to the paralysis and death of insects [54]. In contrast to the case of many animals, insects are thought to have relatively few (on the order of 10 to 12) nAChR type receptor gene families. In *T. cinnabarinus*, 9 nAChRs unigenes have been identified (Table S9).

Acetylcholinesterase (AChE) is a very important neurotransmitter hydrolase that maintains the in vivo cholinergic nerve impulses and is an important target of organophosphate and carbamate insecticides. Inhibition of AChE by insecticides could lead to the accumulation of acetylcholine in the synapses and excessive levels of acetylcholine block depolarization, thus inhibiting normal nerve conduction and ultimately leading to the death of insects. It has been found that changes in AChE are one of the important mechanisms for insect resistant to organophosphate and carbamate insecticides. Many single amino acid substitutions can be detected in the AChE gene, which either act alone or as combination to decrease the sensitivity of AChE to insecticide [24], [25]. One of the CCEs was identified (clade J in Figure 8) in *T. cinnabarinus* and it belonged to the AChE class (Table S7).

The GABA receptors also belong to the super family of cys-loop neurotransmitter receptors. GABARs are the main target for the phenylpyrazole insecticides (such as fipronil), abamectin and cyclopentyl diene insecticides [55]. It has been reported that the mechanism underlying GABAR target resistance is the replacement of one alanine by serine or glycine in the open reading frame. This alanine plays a very important role in the binding between GABARs and insecticides, and the substitution of this alanine causes insects to become resistant to insecticides [55]. Insect GABA receptors are divided into three classes. These are known to be encoded by the Rdl, Grd, or Lcch3 gene. Interestingly, most insect genomes sequences contain only one Rdl orthologues, however we found 3 Rdl orthologues, 2 GABA-A receptors and 3 GABA-B receptors in *T. cinnabarinus* (Table S10).

Sodium channel is the main target of DDT and pyrethroid insecticides. Pyrethroids can interfere with gating kinetics of sodium channels, slowing inactivation during membrane depolarization and extending the sodium ion current, and thus can cause repetitional discharges and blocked nerve conduction [56], [57]. Many Studies have shown that nonsynonymous mutations in the sodium channel involve in insecticide resistance. Our previous study of CSM (GenBank accession number: GU196305) has showed that mutations on sodium channel were associated with fenpropathrin resistance [58]. In this study, we found 7 transcripts hit against sodium channel with 100% accuracy (Table S11).

ATP synthase (ATPase) is one of the targets of beta-cypermethrin. It has been found that target resistance caused by reduced Na^+^-K^+^-ATPase and Ca^2+^-ATPase sensitivities is one of the important mechanisms that involve in beta-cypermethrin resistance of insects [59]. Studies have found that the toxicological mechanisms of pyrethroid insecticides are closely related to the Na^+^-K^+^-ATPase in insect nervous system [60]. Two Na^+^-K^+^- ATPase and 4 Ca^2+^- ATPase were identified from our CSM transcriptome assembly analysis (Table S12).

Cytochrome b (Cytb) is an important class of redox proteins in organisms. It locates in the electron transfer chain, and participates in a series of oxidation-reduction reactions of the living body, including the NADP dependent fatty acid desaturation, oxidation-reduction reactions catalyzed by cytochrome P450 and redox reactions of methemoglobin. Cytochrome b (Cyt b) was newly reported to be the alternative target for acaricide bifenazate [26], [27]. We identified 12 Cyt b sequences from the CSM transcriptome assembly analysis (Table S13).

## Conclusions

We obtained 45,016 contigs and 25,519 unigenes by sequencing the CSM transcriptome. BLAST was used to search the nr, SwissProt, the Clusters of Orthologous Groups (COGs), Kyoto Encyclopedia of Genes and Genomes (KEGG), Gene Ontology (GO) databases and *T. urticae* genome, from which 15,167 unigenes were annotated. These assembled unigenes were used for the identification of the CSM genes associated with insecticide resistance. Totally, 53 P450-related genes, 23 CarEs-, 22 GSTs-, 8 GABA receptor-, 1 AchE- 1 sodium channel-, 12 Cyt b-, 7 GluCls-, 6 ATPase- and 9 nAChRs-related genes from the CSM transcriptome were identified. These gene categories have been reported to be related to insecticide resistance.

We, for the first time, employed RNA-seq to provide a comprehensive identification of the critical elements that may involve in the development of insecticide resistance in CSM. This study utilized for the first time high-throughput sequencing technology to investigate the CSM transcriptome. Although most of the unigenes are not full length, they will greatly improve our understanding of CSM at the molecular level, and the large amount of gene sequences laid a very important foundation for the future investigation of the CSM genes with either known or unknown function.

## Materials and Methods

### Ethics Statement

The laboratory population of Carmine spider mite (CSM), *T. cinnabarinus* was first collected from the field of Beibei District, Chongqing mulicipality directly under the central government, China and no specific permissions were required for these collection activities because this mite is a kind of agriculture-harmful pest and distributes worldwide. We confirm that the field collection did not involve endangered or protected species.

### Sample Preparation

The laboratory CSM population was derived by transferring the CSMs growing on cowpea (*Vigna sesquipedalis* Koern) leaves from the field of Beibei District, Chongqing mulicipality, China to fresh potted cowpea leaves in the lab, and had been raised in artificial climate chamber for 14 years without any insecticide. The rearing conditions were: 26±1°C temperature, 35% to 55% humidity, and 14∶10 (L: D) photoperiod.

Water was added to 60 Petri dishes with a diameter of 12 cm, and two pieces of sponge 4 cm×3 cm×2 cm in size were placed in each dish. Each sponge block was covered with a thin layer of absorbent cotton to increase the water absorption of the sponge, and a piece of filter paper with a size matched exactly with the sponge was placed on top of the sponge wrapped with absorbent cotton. Fresh leaves of cowpea seedling, which were slightly smaller than the filter paper, were collected, cleaned with water, wiped dry, and then placed on the filter paper with the top of leaves facing down. Each leaf was then transferred with 20 female adult mites. The female mites were allowed to lay eggs for 48 h, and then removed. The number of eggs was recorded, and each leaf contained around 200 to 400 eggs. Subsequently, 2-day-old eggs, 0.5-day-old larva, 1-day-old nymphs and 3-day-old female adult and male adult mites were collected.

### RNA-seq and Sequence Information

We collected 4000 eggs, 2000 larva, 1000 nymphs, 800 female adult mites, and 1000 male adult mites, which were placed in a mortar, mixed with liquid nitrogen and fully grounded. The RNeasy plus MicroKit kit (Qiagen GmbH, Hilden, Germany) was used to extract the total RNA of mites at each life stage in accordance with the product manual. The total RNA concentration was above 400 ng/ul, and the total amount RNA of each stage was greater than 20 ug. The quality of the RNA sample was verified by ensuring that the OD260/280 was within the range of 1.8 to 2.2 as measured by the NanoVue UV-Vis spectrophotometer (GE Healthcare Bio-Science, Uppsala, Sweden), and the RNA integrity number (RIN) was greater than or equal to 7 (RIN value was measured by the BGI-Shenzhen). In addition, qualified samples also had a 28S to 18S rRNA ratio above 1.0 as measured by 1% agarose gel electrophoresis.

The qualified RNA sample was send to BGI (Beijing Genomics Institute, China) for Illumina sequencing and standardized analyzing (include estimation of data output, composition and quality assessment of sequencing data, results of the assembly, functional annotation of Unigene, GO classification, and Pathway enrichment analysis). Briefly, after extracting the total RNA from samples, magnetic beads with Oligo (dT) were used to enrich eukaryotic mRNA. The fragmentation buffer was added to break mRNAs into short fragments. The mRNA were used as the template to synthesize the first strand cDNA using random hexamers, followed by synthesis of the second strand cDNA by adding buffer, dNTPs, RNase H and DNA polymerase I. The cDNA was purified by the Qiaquick PCR kit and eluted with the EB buffer, followed by end repair, addition of poly (A) and ligation of the sequencing adaptor. The size of the fragments was selected by agarose gel electrophoresis, followed with PCR amplification. The constructed sequencing library was sequenced using the Illumina HiSeq 2000.

### *De* *novo* Assembly

**Figure 9:**
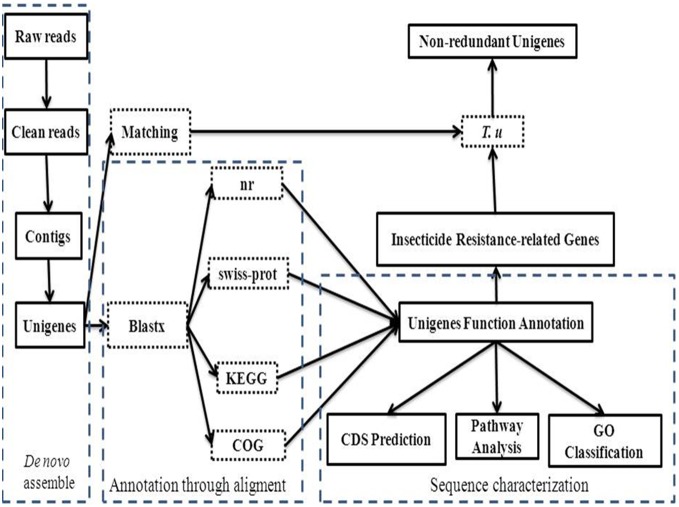
Flow chart of methods used for data analysis.

Figure 9 provides a flow diagram of a computational procedures used for this study. Before being assembled, the clean reads generated by Illumina sequencing were filtered to remove low quality reads from raw reads. First, *de novo* transcriptome analysis of the clean reads was carried out using the short-read assembly program Trinity [61] to generate longer fragments, which were called contigs. Next, the reads were mapped back to contigs. Finally, Trinity connects the contigs, and gets sequences that cannot be extended on either end. Such sequences are defined as Unigenes.

### Transcript Annotation

All *de novo* assembled unique transcripts were compared to protein databases including nr, KEGG, COG and *T. urticae* genome sequence information (http://bioinformatics.psb.ugent.be/webtools/bogas/overview/Tetur) using the Blastx algorithm with a significant cut-off of E-value <10^−5^. The best matches were used to identify coding regions and to determine the sequence direction. The functional annotations of the sequences were predicted using nr database, then Blast2GO was used to get GO annotation of Unigenes [62]. WEGO software was used to do GO functional classification for all unigenes and to understand the distribution of gene functions of the species from the macro level. KEGG is a database that is able to analyze gene product during metabolism process and related gene function in the cellular processes. With the help of KEGG database, we can further study genes’ biological complex behaviors, and by KEGG annotation we can get pathway annotation for unigenes. Unigenes are firstly aligned by blastx (E-value <10^−5^) to protein databases in the priority order of nr, Swiss-Prot, KEGG and COG. When all alignments are finished, proteins with highest ranks in blast results are taken to decide the coding region sequences of Unigenes, the coding region sequences are translated into amino sequences with the standard codon table. So both the nucleotide sequences (5′ - 3′) and amino sequences of the unigene encoding region are acquired. Unigenes that cannot be aligned to any database are scanned by ESTScan, producing nucleotide sequence (5′ - 3′) direction and amino sequence of the predicted coding region.

### Protein Comparison

The entire assembled transcript dataset was used to search for the best hit homologous proteins (BLASTX cut-off e-value 1.0E-5) in the *T. urticae* proteomes. Ortholog prediction was done by performing BLASTX and TBLASTN bidirectional comparisons between *T. cinnabarinus* and *T. urticae* (e value 1.0E-5) to identify the hits within the two species.

To identify the proportion of the core eukaryotic genome covered by the *T. cinnabarinus* transcriptome, we used HMM profiles corresponding to the 458 core eukaryotic proteins as provided by the CEGMA algorithm [31]. Local HMMER3 searches [63] were calibrated using the *T. urticae* core eukaryotic protein validated dataset consisting of 448 sequences [22].

### Identification of Genes Related to Insecticide Resistance

To identify the sequences encoding genes related to insecticide resistance, such as detoxification enzymes (GSTs, CarEs and P450) and insecticide targets (IGluRs, AChE, GABA receptor, sodium channel, Cytochrome b, ATP synthase and nAChRs), sequences encoding potential pesticide-related genes (>180 bp) were identified using the blastx program in the NCBI database with a cut-off E-value <10^−5^. Among the unigenes shown to contain sequences related to insecticide resistance, some corresponded to the same genes. In these cases the transcripts were advances to a filter to identify different isoforms and transcripts based on being mapped in the *T. urticae* genome database. The unique transcripts mapped in the same blast results or with high homology to one another were eliminated as allelic variants or as different parts of the same gene. In such cases, the longer ones were adopted. PhyML3.1 software [64] was used to analyze the phylogenetic relationships between interested genes with the related genes of other species, especially with *T. urticae*, to make a prediction of their classification and homology. A maximum likelihood analysis was used to create phylogenetic trees of resistance-related genes. Positions containing alignment gaps and missing data were eliminated with pairwise deletion. Bootstrap analysis of 500 replication trees was performed to evaluate the branch strength of each tree.

### Positive Selection Analysis of Metabolic Enzyme

*T. cinnabarinus* orthologs of detoxification genes (P450s, GSTs, CCEs) in *T. urticae* were identified based on our phylogenetic analyses. Pairs having a bootstrap value greater than 90% and alignment length greater than 500nt were selected for further analysis. Pair-wise amino acid alignments of the region in common between two orthologs were conducted using Muscle 3.8.31[65]. The amino acid sequences were back-translated to nucleotide sequences and used for the estimation of the pairwise non-synonymous (dN) and synonymous (dS) substitution rates using MEGA 5.05 [66]. The Jukes-Cantor distance model with the modified Nei-Gojobori method was used. The pair-wise ratios of dN/dS (ω) were calculated and used to investigate if *T. cinnabarinus* sequences were evolved under positive (ω>1) or negative, purifying (ω <1) selection or neutrally (ω = 1) compared to the corresponding sequences of *T. urticae.*

## Supporting Information

Table S1 The result of aligning *T. cinnabarinus* transcriptome with *T. urticae* genome CDS database.
(XLSX)

Table S2 The result of aligning *T. cinnabarinus* transcriptome with *T. urticae* genome proteins database.
(XLSX)

Table S3 Alignment of assembled unigenes to proteins in the CEGMA database, as determined by a BLASTx search.
(XLSX)

Table S4 Distribution of KEGG functional annotation of the CSM transcriptome.
(DOCX)

Table S5 Predicted insecticide resistance-related P450 transcripts. Length: sequences length of the unigene. Identity: seqeunce identity of the alignment to the *T. urticae* protein. *Tu* ID: ID of *T. urticae (Tu)* genome.
(XLSX)

Table S6 Predicted insecticide resistance-related GST transcripts. Length: sequences length of the unigene**.** Identity: seqeunce identity of the alignment to the *T. urticae* protein. *Tu* ID: ID of *T. urticae (Tu)* genome.
(XLSX)

Table S7 Predicted insecticide resistance-related CarEs and AChE transcripts. Length: sequences length of the unigene**.** Identity: seqeunce identity of the alignment to the *T. urticae* protein. *Tu* ID: ID of *T. urticae (Tu)* genome.
(XLSX)

Table S8 Predicted insecticide resistance-related GluCls transcripts. Length: sequences length of the unigene**.** Identity: seqeunce identity of the alignment to the *T. urticae* protein. *Tu* ID: ID of *T. urticae (Tu)* genome.
(XLSX)

Table S9 Predicted insecticide resistance-related nAChRs transcripts. Length: sequences length of the unigene. Identity: seqeunce identity of the alignment to the *T. urticae* protein. *Tu* ID: ID of *T. urticae (Tu)* genome.
(XLSX)

Table S10 Predicted insecticide resistance-related GABA recceptor transcripts. Length: sequences length of the unigene**.** Identity: seqeunce identity of the alignment to the *T. urticae* protein. *Tu* ID: ID of *T. urticae (Tu)* genome.
(XLSX)

Table S11 Predicted insecticide resistance-related VGSC transcripts. Length: sequences length of the unigene**.** Identity: seqeunce identity of the alignment to the *T. urticae* protein. *Tu* ID: ID of *T. urticae (Tu)* genome.
(XLSX)

Table S12 Predicted insecticide resistance-related calcium and sodium- potassium ATPase transcripts. Length: sequences length of the unigene**.** Identity: seqeunce identity of the alignment to the *T. urticae* protein. *Tu* ID: ID of *T. urticae (Tu)* genome.
(XLSX)

Table S13 Predicted insecticide resistance-related Cyt b transcripts. Length: sequences length of the unigene**.** Identity: seqeunce identity of the alignment to the *T. urticae* protein. *Tu* ID: ID of *T. urticae (Tu)* genome.
(XLSX)
