# Supplementary material for: Analysis of Insecticide Resistance-Related Genes of the Carmine Spider Mite Tetranychus cinnabarinus Based on a De Novo Assembled Transcriptome
Source: PLoS One. 2014 May 15;9(5):e94779. doi: 10.1371/journal.pone.0094779 (PMC4022505; doi:10.1371/journal.pone.0094779)
Supplement: Table S4 — Distribution of KEGG functional annotation of the CSM transcriptome. (DOCX) [file pone.0094779.s004.docx]

**Table S4** Distribution of KEGG functional annotation of the CSM transcriptome

| **#** | **Pathways** | **Amount of Unigene** | **Percentage** | **Pathway ID** |
| --- | --- | --- | --- | --- |
| 1 | Metabolic pathways | 1,529 | 13.24% | ko01100 |
| 2 | Pathways in cancer | 439 | 3.80% | ko05200 |
| 3 | Lysosome | 400 | 3.46% | ko04142 |
| 4 | Focal adhesion | 379 | 3.28% | ko04510 |
| 5 | Amoebiasis | 338 | 2.93% | ko05146 |
| 6 | Regulation of actin cytoskeleton | 325 | 2.82% | ko04810 |
| 7 | RNA degradation | 322 | 2.79% | ko03018 |
| 8 | RNA transport | 305 | 2.64% | ko03013 |
| 9 | Huntington’s disease | 303 | 2.62% | ko05016 |
| 10 | MAPK signaling pathway | 293 | 2.54% | ko04010 |
| … | … | … | … | … |
| 237 | Polyketide sugar unit biosynthesis | 2 | 0.02% | ko00523 |
| 238 | Biotin metabolism | 2 | 0.02% | ko00780 |
| 239 | Allograft rejection | 1 | 0.01% | ko05330 |
| 240 | D-Arginine and D-ornithine metabolism | 1 | 0.01% | ko00472 |
| 241 | Graft-versus-host-disease | 1 | 0.01% | ko05332 |
|  | Total Unigene number | 11,545 | 100% | - |
|  | Total Pathways number | 241 | - | - |
